# Expectancy to Eat Modulates Cognitive Control and Attention Toward Irrelevant Food and Non-food Images in Healthy Starving Individuals. A Behavioral Study

**DOI:** 10.3389/fpsyg.2020.569867

**Published:** 2021-01-13

**Authors:** Sami Schiff, Giulia Testa, Maria Luisa Rusconi, Paolo Angeli, Daniela Mapelli

**Affiliations:** ^1^Medical Clinic 5, Department of Medicine, School of Medicine and Surgery, University of Padova, Padua, Italy; ^2^Department of Human and Social Sciences, University of Bergamo, Bergamo, Italy; ^3^Department of Psychiatry, University Hospital of Bellvitge-IDIBELL, Barcelona, Spain; ^4^Department of General Psychology, University of Padova, Padua, Italy

**Keywords:** expectancy, cognitive control, visual attention, Simon task, food, reward sensitivity

## Abstract

It is thought that just as hunger itself, the expectancy to eat impacts attention and cognitive control toward food stimuli, but this theory has not been extensively explored at a behavioral level. In order to study the effect of expectancy to eat on attentional and cognitive control mechanisms, 63 healthy fasting participants were presented with an affective priming spatial compatibility Simon task that included both food and object (non-food) distracters. The participants (*N* = 63) were randomly assigned to two groups: an “immediate expectancy” group made up of participants who expected to eat immediately after the task (*N* = 31; females = 21; age = 26.8 ± 9.6) and a “delayed expectancy” cohort made up of individuals who expected to eat a few hours later (*N* = 32; females = 21; age = 25.0 ± 8.0). Slower reaction times (RTs) toward the food and non-food distracters and a more pronounced effect on the RTs in the incompatible condition [i.e., the Simon effect (SE)] were noted in both groups. The effect of the food and non-food distracters on the RTs was more pronounced in the immediate with respect to the delayed expectancy group. The magnitude of the SE for the food and the non-food distracters was also greater in the immediate with respect to the delayed expectancy group. These results seem to indicate that when the expectancy to eat is short, the RTs are delayed, and the SE is more pronounced when food and non-food distracters are presented. Instead, when the expectancy to eat is more distant, the distracters have less of an effect on the RTs and the correspondence effect is smaller. Our results suggest that the expectancy to eat can modulate both attention orienting and cognitive control mechanisms in healthy fasting individuals when distracting details are competing with information processing during goal directed behavior.

## Introduction

Although food can be considered a primary reward (Berridge, 1996), it is nevertheless essential for our survival. It is widely recognized that food deprivation increases the reinforcement value of a food reward (Raynor and Epstein, 2003; Epstein and Leddy, 2006), suggesting that an individual’s metabolic state can modulate subjective motivation and the desire to eat. As food salience seems to be regulated by energy balance and hedonic hunger interaction, these mechanisms may affect how we process environmental cues (Benarroch, 2010; Berthoud, 2011).

Given their salience for survival under specific metabolic conditions, food stimuli may trigger motivational approach processes including allocation of attentional resources (i.e., food-related attentional-bias) and cognitive control toward food stimuli (Nijs et al., 2010; Testa et al., 2020). Both attentional bias and cognitive control in the presence of food-related stimuli have been shown to be intensified in healthy individuals by a variety of conditions, including food and sleep deprivation and mood modulation (Mogg et al., 1998; Stockburger et al., 2009; Forestell et al., 2012; Loeber et al., 2013; Sänger, 2019). Other factors that have been shown to modulate the magnitude of food-related attentional bias and cognitive control toward food (i.e., response inhibition and interference control) seem to be conditioned by an individual’s characteristics, including personality traits (e.g., attentional impulsivity) (Hou et al., 2011; Jasinska et al., 2012), eating styles such as eating in response to external food cues (i.e., external eating), and weight status (Castellanos et al., 2009; Werthmann et al., 2011; Yokum et al., 2011; Hendrikse et al., 2015; Carbine et al., 2018; Testa et al., 2020).

Neuroimaging research in healthy participants has shown that food stimuli are processed in the brain by an extended network encompassing primary sensory areas depending on the sensory modality (e.g., visual, olfactory) regions involved in reward processing such as the insula and the orbitofrontal cortex (OFC) and areas involved in control of attention and cognition such as the lateral prefrontal cortical regions (see for systematic meta-analysis: van der Laan et al., 2011; Huerta et al., 2014). Food-deprived individuals show enhanced activity in reward-related brain areas (LaBar et al., 2001; Porubská et al., 2006; Führer et al., 2008; Siep et al., 2009), while satiated participants show enhanced activity of the lateral prefrontal areas [e.g., the dorsolateral prefrontal cortex (DLPFC)] in Gautier et al. (2001), Smeets et al. (2006), Thomas et al. (2015). DLPFC activation has also been associated with higher levels of self-control over food choices, suggesting that it is involved in controlling food intake (Hare et al., 2009; Hollmann et al., 2012).

Another factor that seems to affect food-related processing in the brain is the anticipation of receiving an immediate food reward. It has been posited that the expectancy to receive a food-related gratification increases the activation of those brain regions, such as the OFC, the dopaminergic midbrain, the amygdala, and the striatum that are involved in reward processing (O’Doherty et al., 2002). It has nevertheless been reported that in monkeys the expectancy of receiving a reward after a particular response is associated with activity in the DLPFC (Watanabe, 1996). These data suggest that expectation of a reward modulates brain areas involved in cognitive control and reward processing, possibly facilitating goal-directed behaviors concordant with the incentive value of the contingent reward expected (Berridge, 1996; Watanabe, 1996).

Malik et al. (2011) set out to investigate the immediate as well as delayed effects of the expectancy to eat on human information processing of food and non-food images. The fasting participants participating in their study were instructed to look at images of food and scenery during two different functional magnetic resonance (fMRI) sessions. In one session, the participants were informed that they could expect to eat immediately after the session (the immediate expectancy condition); in the other, they were informed that they could expect to eat a few hours later (the delayed expectancy condition). The results showed that the food images compared with scenery images yielded bilateral activation in the visual areas as well as in the left insula and amygdala in both food expectancy conditions. In the delayed expectancy one, however, the left DLPFC, the hippocampus, and the putamen were additionally activated, while in the immediate expectancy condition, the right OFC activity was enhanced. These data suggest that temporal information regarding immediate or delayed eating affects the salience of food-related stimuli in starving individuals, modulating the activity of the brain areas involved in reward processing and cognitive control.

In a study investigating starving individuals, it was found that the expectancy to receive a food reward influenced the early orientation of attention toward food pictures (i.e., there was a gaze direction bias) (Hardman et al., 2014). Generally speaking, however, there is a paucity of studies investigating how the expectancy to eat affects orienting attention attentional bias and cognitive control.

A novel affective version of the Simon task using food and non-food distracters was recently developed to investigate their effects on cognitive control and attention orienting in starving normal-weight and severe obese individuals (Testa et al., 2020). A study using the new Simon task reported that with respect to a condition without distracters (i.e., neutral condition), distracting images interfere with orienting of attention (i.e., attentional bias) delaying the overall response speed and cognitive control by slowing down reaction times (RTs) when incongruent spatial information competed for response selection (i.e., cognitive control bias). In addition, severely obese individual showed a larger cognitive control bias for food images compared to controls, and a linear relationship was found between subjective hunger perception and the RTs registered during the spatial incongruent condition in the presence of the food images in both the normal weight and severely obese participants (Testa et al., 2020).

The original Simon task, which was devised to study the response selection phase of information processing, typically involves participants who are asked to respond to a task-relevant stimulus (a color or an image) as quickly as possible by pressing the same color coded button that may be on the right or left. Another task-irrelevant feature is also presented. Researchers have found that RTs are faster when the task-relevant stimulus and the response position correspond, meaning they are on the same side (i.e., the corresponding condition) than when they are not (i.e., the non-corresponding condition). This correspondence (faster responses for spatial correspondence, slower responses for non-spatial correspondence) has been called the Simon effect (SE) (Simon and Rudell, 1967; Nicoletti and Umiltà, 1994; Lu and Proctor, 1995). It has been posited that the SE is determined by a conflict between two pathways: the fast direct automatic pathway activating the response spatially corresponding with the stimulus location and the slow indirect controlled pathway activating the appropriate response depending on task demands (Cohen et al., 1990; Tagliabue et al., 2000; Ridderinkhof, 2002).

As the newly developed affective Simon task seemed suitable to study food-related attentional bias and its interference with cognitive control mechanisms, we used it to evaluate how immediate or delayed expectancy to eat can modulate RTs and interference control in fasting individuals. The healthy volunteers who were enrolled were asked to fast, and on the scheduled day, they were randomly assigned to one of two groups. Those assigned to the first group were advised that they would be given something to eat immediately after the experimental session; those assigned to the second one was advised that they would be given something to eat a few hours later. During this Simon task, food and non-food images are able to interfere or bias at two different levels of information processing: at the time attention is being oriented and during response selection. With regard to the former, the cues are expected to affect the time required to orient attention toward task relevant information, delaying overall RTs when distracting (in particular, photos of food) images are presented. With regard to the second, they are expected to affect cognitive control functions when conflicting spatial information is presented.

In the light of these considerations and the knowledge presently available on mechanisms modulating or biasing orienting attention and/or cognitive control when motivationally salient but task-irrelevant images are presented together with task-relevant stimuli, we designed an experiment and formulated different hypotheses. First, we expect to replicate findings of our previous work by Testa et al. (2020) showing the effect of food/non-food distracters on orienting attention (i.e., delayed RTs compared to the neutral condition) and a food specific effect on cognitive control (i.e., larger SE with food distracter) in starving individuals.

Second, we hypothesis that food and non-food images have a more pronounced effect on RTs (i.e., delaying them) in the immediate expectancy group with respect to the delayed one which would suggest a modulation of expectancy on orienting attention bias. Third, we expect that task-irrelevant distracters have a more pronounced interference on cognitive control (i.e., the magnitude of the SE) in participants expecting to eat shortly with respect to those expecting to wait; this would suggest a modulation of expectancy of cognitive control during response selection. The effect of expectancy over cognitive control could be food-specific or more general in presence of task-irrelevant distractors (both food and non-food).

## Materials and Methods

### Study Participants

The sample size could not be calculated *a priori* due to insufficient information during the planning of the study; thus, a convenient sample of 64 right-handed healthy individuals was chosen. The participants were mainly graduate and post-graduate students from the Universities of Padova and Bergamo who volunteered to participate (Table 1: participants’ socio-demographic and anthropometric variables). A clinical interview was conducted to assess the history or the presence of neurological and/or psychiatric condition. The study’s exclusion criteria were neurological diseases, psychiatric disorders, and being younger than 18 or older than 65. All the participants received a full explanation of the experimental procedure we were using and were asked to sign a consent form. The study was performed in accordance with the Helsinki Declaration (Editors, 2004) and approved by the local Ethical Committee (Padova-University Hospital ethical committee Prot. N.: 3067/AO13).

**TABLE 1:** Mean (standard deviation) values of the socio-demographic and anthropometric variables of the entire group and of the two sub-groups (immediate and delayed expectancy).

	Male/female	Age (years)	Education (years)	Height (cm)	Weight (kg)	BMI (kg/m^2^)
All the participants (*N* = 63)	21/42	25.9 (8.8)	13.9 (2,6)	169 (8.00)	61.2 (10.1)	21.2 (2.5)
Immediate Group (*N* = 31)	10/21	26.8 (9.6)	13.7 (2.7)	170 (8.00)	60.0 (8.8)	20.7 (2.0)
Delayed Group (*N* = 32)	11/21	25.0 (8.0)	14.2 (2.4)	169 (9.00)	62.4 (11.2)	21.7 (2.8)

*The *t*-tests for independent groups did not uncover any differences between them.*

### Procedure

All the participants were instructed to fast for 6 h prior to the experimental session which, in all cases, was scheduled at the same time of the day (12–2 p.m.). Adherence to this instruction was tested asking to them the time of their last meal and if they had eaten something other before coming to the laboratory. Each participant filled out a series of questionnaires (listed below) and subjective ranking of hunger/satiety/desire to eat at the beginning of the session and was then randomly assigned to one of two groups. These self-report measures were adopted to exclude the confounding effect of group differences in impulsivity, eating-related attitudes, and subjective perception of hunger/satiety/desire to eat. Those in Group 1 were informed that they would eat immediately after the task (i.e., immediate expectancy group); those in Group 2 were informed that they would eat 2 h later (i.e., delayed expectancy group).

### Material

#### Affective Simon Task (See Figure 1)

The experimental setting was a dimly lit room. Each participant was seated in front of (58 cm away from) a 15-inch CRT computer screen. The task consisted of 480 experimental trials presented in four blocks, each consisting in 120 trials. A practice block of 42 trials preceded the beginning of the real session. The participant was reminded by a message appearing on the screen before he/she read the instructions for the task and at the beginning of each block of trials that that he/she would be able to eat immediately (if he/she was in Group 1) or 2 h later (if he/she was in Group 2).

**FIGURE 1:**
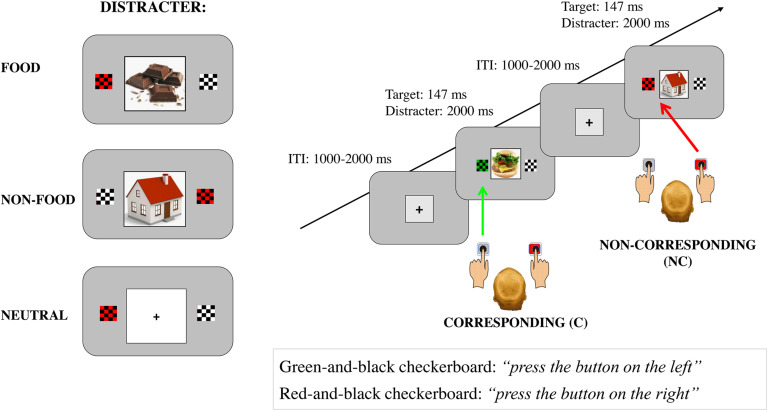
A schematic drawing of the trials using a Simon task and examples of both corresponding (C) and non-corresponding (NC) conditions (on the right) and the three types of distractors presented during the task: a food item, an object, and a neutral distractor (on the left).

Each trial started with a central black fixation cross subtending 0.5° of visual angle, displayed on a light gray background. The fixation cross was surrounded by a black square perimeter with the side subtending 3° of visual angle. After a variable interval, ranging from 2000 to 3500 ms, the target stimuli were presented at an eccentricity of 4.5° of visual angle on the left or right of the fixation cross for 147 ms. The target stimuli were 4 × 4 red-and-black or green-and-black checkerboards subtending 1.48° of visual angle. A 4 × 4 black-and-white checkerboard was presented together with the target as contralateral filler. A central distracter (a cross) was also displayed inside the square for 2000 ms. The distracters consisted of images of food, objects, or a black cross projected on a white background (neutral condition). The duration of the inter-trial intervals ranged from 1000 to 2000 ms. Ten food and 10 non-food images (objects) were selected from a validated dataset (Blechert et al., 2014)^1^.

The participants were instructed to keep their eyes on the screen and to respond to the task-relevant stimulus as quickly and accurately as possible. Half of the participants were instructed to press the left button (the letter “Z” of the keyboard) with their left index finger if the target was the red-and-black checkerboard, and the right button (the letter “M”) with their right index finger if it was the green-and-black one, independently of its spatial position.

These instructions were inverted for the other half of the participants. The three types of distracters (a piece of food, a non-food object, and a cross on a neutral white background which we considered a neutral condition) were presented in half of the cases with corresponding color/location responses and in the other half with non-corresponding color/location responses. The RTs and the accuracy of the responses of each participant for each trial were registered. Individual RTs and accuracy (i.e., probability of correct response) in the different task conditions were screened for outliers, given a cutting point of 2 standard deviations (SD) from the mean response value (conservative threshold). The data of one participant whose percentage of correct responses was lower than two SD of the mean accuracy rate were not included in our analyses.

To control for a speed accuracy trade-off, the mean RTs adjusted for response accuracy [adjRTs = RTs/p (correct response)] were calculated. Data are reported as means ± SD.

#### Self-Report Measures

The Yale Food Addiction Scale (Innamorati et al., 2015) was used to investigate additive eating patterns, the Binge Eating Scale (BES; Gormally et al., 1982) was used to investigate the presence of binge eating behavior, the Power of Food Scale (PFS; Lowe et al., 2009) was used to investigate the attraction to food, the Dutch Eating Behavior questionnaire (DEBQ; van Strien et al., 1986) was used to assess emotional, external, and restrained eating patterns, and the Eating Attitude Test 26 Item (EAT-26; Garner et al., 1982) was used to investigate eating disorders. The Barratt Impulsiveness Scale (BIS-11; Fossati et al., 2001) and the Behavioral Inhibition System/Behavioral Activation System (BIS/BAS; Carver and White, 1994) were used to measure two motivational systems.

The participants’ subjective levels of hunger, satiety, and desire to eat were rated using Likert scales ranging from −5 (max) to 5 (min).

### Data Analysis

A series of *t*-tests for an independent group were first performed to exclude differences in the participants’ socio-demographic and anthropometric variables (i.e., age, years of education, height, weight, and body mass index = kg/m^2^).

To test and corroborate previous findings on the effect of food and non-food distractors over orienting attention (i.e., adjRTs) and cognitive control (i.e., magnitude of SE), in starving individuals, we first run 2 × 3 repeated measures ANOVAs with correspondence (C vs NC) and the type of distracter (food, object, and neutral) as within participants independent variable factors.

Then, to test the effect of expectancy over the orienting attention and cognitive controls biases induced by food or non-food distracters, RTs in the C and the NC trials for the food and object distracters were separated from those for the neutral condition (i.e., C_food – C_neutral; NC_food – NC_neutral; C_object – C_neutral; NC_object – NC_neutral), and second 2 × 2 × 2 repeated measures ANOVA was run with the group as between individual variable (immediate vs delayed), and the correspondence (C, NC) and the type of distracters (food and object) as participants individual variables.

The effect size was expressed as the partial eta squared and interpreted according to Richardson (2011) (<0.06 low; 0.06–0.14 moderate; >0.14 high).

## Results

The *t*-tests used to analyze the participants’ socio-demographic and anthropometric variables did not uncover any differences in the ages, years of education, height, weight, or body mass index variables of the two groups (Table 1). *T*-tests applied on self-report measures of subjective hunger/satiety/desire to eat, eating attitudes and traits of impulsivity did not show any significant difference between the two groups (see Supplementary Tables S1–S3 for a detailed description of the results).

**FIGURE 2:**
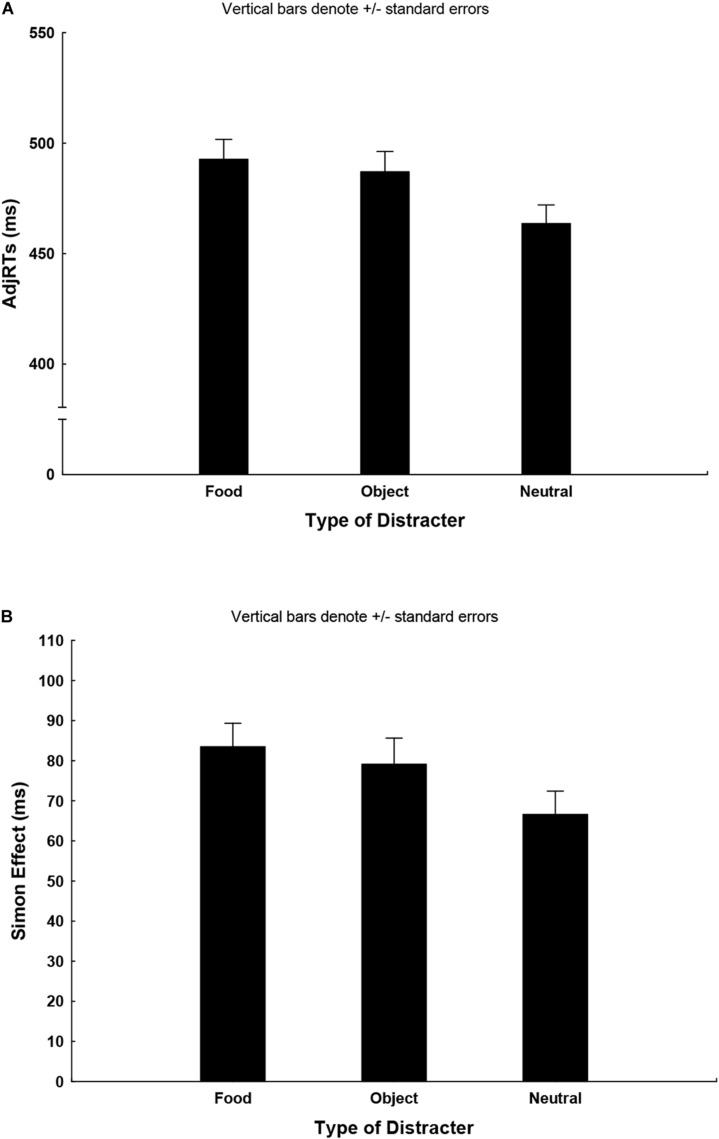
**(A)** The graph shows the mean adjRTs for the three distracters (i.e., the food, the non-food, and the neutral images) of all the participants. The ANOVA uncovered the main effect the type of distracters, revealing that both food and non-food distracters slow down RTs compared with the neutral condition. **(B)** The graph shows magnitude of the Simon effect for the three distracters (i.e., the food, the non-food, and the neutral images). The ANOVA on adjRTs uncovered the interaction between correspondence and the type of distracters, and planned contrast on the magnitude of the Simon effect reveal that only food distracters increase the magnitude of the Simon effect compared to the neutral condition (*p* < 0.009).

The ANOVA on adjRTs showed the significant main effect of the type of distracter: *F*(2,122) = 46.1; *p* = 0.000001; η${}_{p}^{2}$ = 0.43, with slower RTs for both the food and non-food distracters compared to the neutral condition (food: 492 ± 74 ms mean ± SD; object: 487 ± 77 ms; neutral: 463 ± 70 ms; Bonferroni food vs neutral, *p* < 0.00001; object vs neutral, *p* < 0.00001; Figure 2A) and the main effect of correspondence: *F*(1,61) = 219.7, *p* = 0.00001; η${}_{p}^{2}$ = 0.78, showing longer RTs in the NC condition (C: 443 ± 73 ms; NC: 519 ± 76 ms). This result reveals an attentional orienting bias of distracters images (both food and objects) on RTs. An interaction between correspondence and type of distracter: *F*(2,122) = 5.0, *p* = 0.008; η${}_{p}^{2}$ = 0.07 was also detected, with *post hoc* showing longer RTs for the NC with respect to the C trials for all types of distracters. Planned contrast on the SE highlighted a larger magnitude of the SE only for food distracters with respect to the neutral condition (food: 83 ± 46 ms; neutral: 67 ± 46 ms; *p* < 0.009; Figure 2B), depicting a food-specific cognitive control bias in our starving participants.

**FIGURE 3:**
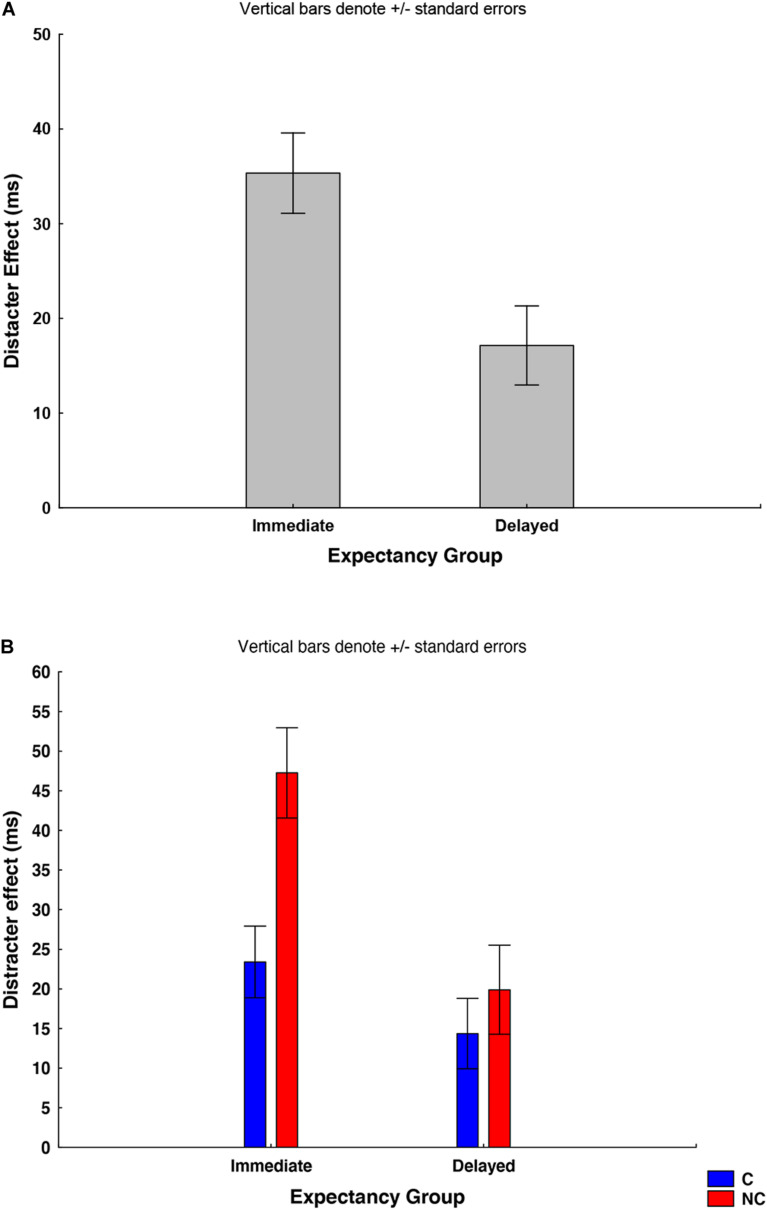
**(A)** The graph shows the “distracter effect” (i.e., the differences in the RTs collected during the food and non-food distracting trials and those collected for the neutral condition). The ANOVA analyzing the two groups (immediate and delayed expectancy) separately uncovered the effect of group, highlighting a significantly higher attention bias produced by the food and non-food images in the immediate expectancy group with respect to the delayed one. **(B)** An interaction between group and correspondence was also found, revealing a significant difference between C (blue bars) and NC trials (red bars) in the immediate expectancy group, but not in the delayed one. *Post hoc p*s < 0.05.

The analysis to the test the effect of expectancy uncovered a significant main effect of group: *F*(1,61) = 9.6, *p* = 0.003; η${}_{p}^{2}$ = 0.13, confirming the larger orienting attention bias due to distracter images in the “immediate expectancy” group (food-neutral: 38 ± 33 ms; object-neutral: 32 ± 33 ms) with respect to the “delayed expectancy” group (food-neutral: 20 ± 31 ms; object-neutral: 14 ± 34 ms; Figure 3A). Interestingly, together with the main effect of correspondence: *F*(1,61) = 13.0, *p* = 0.0006; η${}_{p}^{2}$ = 0.17, revealing a larger effect of food and non-food distracters on the NC condition (33 ± 38 ms) with respect to the C one (21 ± 33 ms), a significant group × correspondence interaction was also detected: *F*(1,61) = 5.04, *p* = 0.03; η${}_{p}^{2}$ = 0.08. *Post hoc* analysis on this later effect revealed a significant difference between C and NC conditions in the immediate expectancy group (NC = 47 ± 36 ms, C = 23 ± 30 ms; *p* < 0.001), but not in the delayed group (NC = 19 ± 36 ms, C = 14 ± 28 ms; *p* < ns). In addition, the effect of distracters in the NC condition was larger in the immediate expectancy group with respect to that in the delayed group (NC immediate group: 47 ± 36 ms, NC immediate group: 19 ± 36 ms; *post hoc p* < 0.001). This result suggests that the bias on cognitive control induced by food and non-food distracters was larger in the immediate with respect to the delayed expectancy group (Figure 3B).

## Discussion

The current study set out to evaluate the effect of the expectancy to eat on orienting attention and cognitive control in the presence of distracting food or non-food images. A modified version of the Simon task was administered to healthy fasting participants who were divided into two groups: those who were told they would be given something to eat immediately after the session and those who were told they would be given something to eat a few hours later.

Results corroborate our hypothesis, showing that immediate/delayed expectancy modulates both orienting attention and cognitive control bias, and these effects seem not to be food specific.

According to our first hypothesis, the participants’ overall RTs were slower during the trials presenting both food and non-food distracters, confirming an orienting attention bias toward task-irrelevant distracting stimuli in starving participants. This is consistent with the findings of our previous work adopting the same task (Testa et al., 2020), and can presumably be explained by the interference of task-irrelevant centralized food and non-food images on those mechanisms involved in visually orienting attention toward lateralized task-relevant information.

One of the most relevant models for the attention system suggests that orienting attention in space is characterized by three partially independent mental operations: (1) engaging, (2) disengaging, and (3) moving (Posner and Petersen, 1990). The results of our trials indicate that distracting images during the visual orientation stage of an affective Simon task may cause an initial engagement of selective attention toward their position, even if it is irrelevant to the task. The participants needed to disengage their attention resources from the central image and move them toward the lateralized stimuli in order to focus on the task-relevant position. This additional, probably time-consuming process may explain why the RTs for the food and non-food distracters were longer than those for the neutral one.

The second hypothesis of a food-specific effect over cognitive control in starving individual was corroborated by the larger SE registered for the food (but not for the non-food) images with respect to the neutral condition. The interference of food on the response selection is possibly linked to the effect of hunger on cognitive control processes. Similarly, a correlation between the magnitude of the SE in the presence of food-distractors and the participants’ subjective hunger perception was previously detected in starving individuals (Testa et al., 2020).

Hunger may have increased the motivational salience of stimuli coming from the external environment affecting the time necessary to process them or to disengage attentional resources. A non-task relevant engagement of cognitive resources especially for food-related stimuli may have enhanced the ipsilateral activation response to the stimulus position, making additional cognitive control resources necessary to select the correct response. A mechanism of this kind would corroborate the findings of behavioral studies carried out in hungry individuals suggesting that hunger has a direct effect on the salience of food cues as it modulates inhibitory control over food-related response selection (Loeber et al., 2012, 2013).

Regarding the effect of expectancy over orienting attention, results indicated a larger distracting effect in the immediate expectancy group compared to the delayed one, which seems not to be specific for food-related stimuli. Thus, the finding suggests that expectancy modulates the efficiency of at least one of the operations involved in orienting attention toward a task-relevant lateralized feature, probably affecting mechanism related to disengagement of attention form irrelevant distracting image. It is possible that selective attention network’s predisposition to potentially salient environmental stimuli is enhanced by the immediate expectancy to eat, a hypothesis that is certainly plausible in evolutionary terms in starving individuals. The effect is probably attenuated in the delayed expectancy group by the larger amounts of attentional resources allocated to task demands in the individuals who must refrain from thinking about food and repress their desire to eat for a longer time. In fact, the distracting effect was smaller. With respect to cognitive control, the interaction between expectancy and correspondence suggests that an immediate expectancy to eat enhances the distracting effect of both food and non-food images on spatial correspondence.

Taken together, these results suggest that the effect of expectancy affects both orienting attention and response selection in an independent but similar manner. Research focusing on cognition (Kornblum et al., 1990; Hommel and Prinz, 1997) describes different sources of conflict depending on the locus of interference, suggesting that there is a distinction between processing stimulus-stimulus (S-S) and stimulus-response (S-R) conflicts. For example, while in Stroop and Flanker tasks, conflict is between different features of the stimuli (i.e., an S-S conflict) competing for the selection of the correct response at a perceptual level, in a Simon task, there is a conflict between stimulus and response locations (i.e., S-R conflict). In this latter case, the spatial position of the stimulus is thought to automatically activate the responding hand ipsilateral to the stimulus position. In this light, expectancy seems to affect both stages of information processing, that is during orienting of attention as well as response selection. In the first case, distracters interfere with the selection of task-relevant information (i.e., the S-S conflict), while in the latter, they probably enhance the automatic activation of the response primed by the irrelevant stimulus position at a premotor level (i.e., S-R conflict).

We have the impression that in our study the expectancy to eat in an immediate as opposed to a delayed future further increased the predisposition of the orienting attention system to be automatically captured by potentially salient cues from the external environment. The delayed or postponed expectancy to eat may have, instead, attenuated the effect of irrelevant distracting images on the orienting attention system, probably via top-down influences from higher order brain areas linked to the control of selective attention (Posner and Petersen, 1990). A similar effect also seems to occur at the response selection stage during which the irrelevant position of the stimulus is thought to prime the hand ipsilateral to the stimulus, in which case the distracting effect of food and non-food images seems to be enhanced by an immediate expectancy and reduced by a delayed one.

The effect of expectancy on orienting attention and cognitive control over response selection could be linked to the measure of time the individual is expecting to wait before receiving a reward, in our case, food. Studies examining inter-temporal decision-making suggest that the tendency to settle for a smaller, immediate reward instead of a larger, delayed one is associated with higher impulsivity (Frederick et al., 2002; Sellitto et al., 2011) and with the activity of those brain areas controlling reward-related behavior, in particular, the OFC, the nucleus accumbens, the ventral tegmental area, the striatum, and the amygdala (Sellitto et al., 2010). The preference for a delayed, larger reward is, instead, associated with cognitive control and the activity of those areas implicated in executive control, in particular, the DLPFC (Figner et al., 2010), and is altered in obese individual (Schiff et al., 2016). These considerations seem to fit quite nicely with our findings. Regardless of individual differences in reward processing or cognitive control, the delayed expectancy to eat seemed to reduce the immediate expectation of receiving a reward, increase allocation of cognitive resources, and reduce impulsive behavior. An immediate expectancy to eat seemed, instead, to increase impulsivity and the need for rapid gratification. Similarly, episodic future thinking (i.e., a vivid mental simulation of future experiences) has been shown to reduce the preference for immediate rewards during a temporal discounting task (Peters and Büchel, 2010). In fact, when episodic future thinking concerns food-related thoughts, it has been found to reduce food intake and snacking in both healthy individuals (Dassen et al., 2016) and in obese patients (Daniel et al., 2013). Another study showed that episodic future thinking techniques reduced impulsive choices and alcohol consumption in alcohol-addicted individuals (Snider et al., 2016). By the same token, our data suggest that a mental projection of a delayed expectancy to eat could reduce impulsivity. Future studies investigating clinical populations characterized by impulsivity (e.g., individuals involved in substance abuse; behavioral addiction; binge eating disorders) may contribute to identifying a new treatment approach to enhance cognitive control toward addiction-related cues.

Malik et al.’s (2011) fMRI imaging study demonstrated a specific activation of the DLPFC for the food with respect to the scenery images in the delayed expectancy to eat condition. Thus, when the participants knew that they would not be eating for an extended period of time, they showed cognitive control in response to food cues. Although we were unable to directly explore brain activity during our own study, food stimuli did not appear to interfere with cognitive control in the participants belonging to the delayed expectancy group in whom we were expecting to detect maximal DLPFC activation. Unlike Malik et al.’s (2011) findings, ours demonstrate that the effect of expectancy on RTs was not food specific, but the differences in the paradigms adopted by the two studies may have rendered them incomparable. The modified Simon task we adopted used food and non-food images as task-irrelevant distracters, and our participants were instructed to focus their attention on the color of the lateralized target in order to carry out the task at hand. In Malik et al.’s (2011) study, participants were involved in a cue-reactivity task requiring only a passive view of the images. The difference in the relevance of the images in the two studies may reflect a different type of activation of the reward system, as has been suggested by another fMRI study (Siep et al., 2009). Another study using eye tracking methodology in fasting individuals likewise reported that expectancy to eat did not produce a specific effect on early orientation of attention toward food cues (Hardman et al., 2014). The findings that are presently available seem to indicate that the expectancy to eat has an effect on general mechanisms of selective attention and cognitive control and does not directly impact food reward systems involved in orienting attention.

These findings must be evaluated in the light of limitations. First, all of the participants were tested in fasting state which may have enhanced food salience leading to a similar interference from food stimuli on cognitive control in both expectancy groups. Examining these mechanisms also in satiated individuals would have permitted us to investigate how the desire to eat and food craving rather than hunger come into play in this interaction. Second, participants adherence to the 6 h of fasting before task execution was not objectively monitored (e.g., isolating them before starting the experiment), which is usually recommended in studies that manipulate hunger and satiety. Third, despite the fact that no group differences were found in the participants’ characteristics according to the questionnaires that were utilized, the study design did not permit us to evaluate the effect of expectancy in highly impulsive individuals. Future studies examining healthy participants with high and low impulsivity traits will be able to explore the interaction of the impulsivity trait with expectancy in modulating orienting attention and cognitive control.

Finally, our data are based entirely on behavioral findings; utilizing both neuroimaging techniques and cognitive control tasks would have permitted us to explore neural activity in different expectancy conditions more directly.

## Conclusion

In conclusion, the current study suggests that temporal expectation (immediate vs delayed) of a reward, in this case food in fasting individuals modulates both orienting attention and cognitive control mechanisms when irrelevant but salient stimuli are present in the environment. The expectancy of receiving a food reward in the immediate future increased the distracting effect and reduced the control of selective attention in the presence of stimuli competing with information processing. Furthermore, the expectancy of receiving a food reward in an immediate future reduced cognitive control in the presence of a spatial interference for response selection, hence increasing impulsivity. On the other hand, the expectancy of receiving a food reward in a more distant future produced, instead, a reduction of the distracting effect and enhanced cognitive control over response selection, leading to lower impulsivity.

These results shed new light on the effects of expectancy on cognitive processing in healthy individuals, and they suggest that selective attention and cognitive control may be manipulated also in clinical populations characterized by high levels of impulsivity, such as obese patients and participants with addictive disorders.

## Data Availability Statement

The raw data supporting the conclusions of this article will be made available by the authors, without undue reservation.

## Ethics Statement

The studies involving human participants were reviewed and approved by the Ethics Committee of the Padua University Hospital. The patients/participants provided their written informed consent to participate in this study.

## Author Contributions

SS conceptualized the study. SS and GT recruited participants and collected, analyzed, and interpreted the data. SS and GT wrote the manuscript, with the support, the suggestions, and the correction of PA, MR, and DM. All authors contributed to the article and approved the submitted version.

## Conflict of Interest

The authors declare that the research was conducted in the absence of any commercial or financial relationships that could be construed as a potential conflict of interest.

## Footnotes

1 Image numbers were: 18, 32, 45, 46, 54, 107, 110, 145, 167, 176, 1008, 1025 1033, 1036, 1044, 1060, 1096, 1081, 1117, 1137.
