# Supplementary material for: Expectancy to Eat Modulates Cognitive Control and Attention Toward Irrelevant Food and Non-food Images in Healthy Starving Individuals. A Behavioral Study
Source: Front Psychol. 2021 Jan 13;11:569867. doi: 10.3389/fpsyg.2020.569867 (PMC7838084; doi:10.3389/fpsyg.2020.569867)
Supplement: Supplementary file 1 [file Table_1.pdf]

**Supplementary Material**

**Table S1:** The mean and standard deviation of the hunger, satiety, and desire to eat values of the whole group of participants and in the two subgroups. The t-tests for independent groups did not uncover any differences between the two groups.

| | All the participants (N =63) | Immediate Group (N= 31) | Delayed Group (N =32) | t-value (df=61) | pvalue |
|---|---|---|---|---|---|
| Hunger | -2.8 (1.7) | -2.3 (1.8) | -2.4 (1.7) | .30 | .75 |
| Satiety | -2.9 (1.9) | -2.6 (1.64) | -2.7 (1.6) | .21 | .82 |
| Desire to eat | -3.6 (1.7) | -2.9 (1.71) | -3.1 (1.4) | .63 | .52 |

**Table S2:** The mean and standard deviation values derived from the questionnaires investigating eating patterns. T-tests for independent groups were adopted to compare immediate and delayed group.

| | All the participants (N =63) | Immediate Group (N= 31) | Delayed Group (N =32) | t-value (df=61) | p-value |
|---|---|---|---|---|---|
| BES | 5.2 (4.6) | 4.9 (5.2) | 5.5 (4.1) | -.51 | .61 |
| EAT-26 | 4.2 (7.2) | 2.5 (3.2) | 5.9 (9.4) | -1.9 | .05 |
| Y-FAS | 1.2 (1.1) | 1.1 (1.0) | 1.3 (1.1) | -.80 | .42 |
| DEBQ - Restrain | 2.1 (0.8) | 1.9 (0.7) | 2.2 (0.9) | -1.7 | .08 |
| DEBQ - Emotional | 1.8 (0.7) | 1.8 (0.7) | 1.8 (0.7) | .26 | .79 |
| DEBQ - External | 2.8 (0.7) | 2.9 (0.6) | 2.7 (0.9) | .78 | .43 |

*Note: BES= Binge Eating Scale; EAT-26 = Eating attitude test -26 items, Y-FAS=Yale Food Addiction Scale; DEBQ= Dutch Eating Behaviour Questionnaire*

**Table S3:** The mean and standard deviation values derived from questionnaires investigating personality traits. T-tests for independent groups were adopted to compare immediate and delayed group.

| | All the participants (N =63) | Immediate Group (N= 31) | Delayed Group (N =32) | t-value (df=61) | p-value |
|---|---|---|---|---|---|
| BIS-11 – Attentional | 15.4 (2.8) | 15.7 (3.1) | 15.7 (2.5) | -1.1 | .31 |
| BIS-11 – Motor | 18.6 (3.9) | 18.6 (4.8) | 18.6 (2.8) | -0.1 | .98 |
| BIS-11 – No Planning | 24.2 (3.6) | 24.8 (4.0) | 23.6 (3.1) | 1.2 | .31 |
| BIS-11 – Total | 58.2 (7.6) | 58.7 (9.5) | 57.6 (5.3) | .57 | .56 |
| BIS – Anxiety | 24.1 (5.0) | 23.0 (5.1) | 25.1 (4.8) | -1.7 | .09 |
| BAS - Reward | 19.6 (3.5) | 20.1 (3.5) | 19.1 (3.5) | 1.1 | 2.6 |
| BAS- Drive | 11.5 (3.0) | 11.8 (2.9) | 11.2 (3.0) | .70 | .48 |
| BAS – Fan | 10.9 (3.5) | 11.1 (3.9) | 10.8 (3.2) | .31 | .75 |
| BAS – Total | 45.5 (12.9) | 46.6 (14.9) | 44.4 (10.6) | .68 | .49 |

*Note: BIS-11: Barrat Impulsiveness Scale; BIS: Behavioural Inhibition Scale; BAS Behavioural Activation Scale*
